# Maladie de Von Recklinghausen révélée par une masse thoracique et des lésions kystiques pulmonaires

**DOI:** 10.11604/pamj.2025.52.72.49440

**Published:** 2025-10-14

**Authors:** Imane Laatfa, Hind Lachraf

**Affiliations:** 1 Service de Pédiatrie 2, Unité de Neuropédiatrie et Maladies Neurométaboliques, Hôpital d'Enfants de Rabat, Rabat, Maroc,; 2 Faculté de Médecine et de Pharmacie de Rabat, Université Mohammed V de Rabat, Rabat, Maroc

**Keywords:** Neurofibromatose type 1, maladie de Von Recklinghausen, masse médiastinale, lésions kystiques pulmonaires, Neurofibromatosis type 1, Von Recklinghausen's disease, mediastinal mass, pulmonary cystic lesions, image in medicine

## Abstract

Neurofibromatosis type 1 (NF1) is an autosomal dominant genetic disorder that affects approximately 1 in 3,000 births. It is mainly characterized by cutaneous manifestations such as café au lait spots, neurofibromas, and axillary or inguinal freckling. Pulmonary involvement is rare (5-20%) and may include cystic or infiltrative lesions. We here report the case of a 13-year-old boy with no significant past medical history who presented with exertional dyspnea, chest pain, fatigue, and dry cough. Physical examination revealed multiple café au lait spots (>10), axillary freckling, and dorsal scoliosis. Chest X-ray showed a right posterior mediastinal mass. A computer tomography scan confirmed a large mediastinal mass (13 x 15 x 12cm) associated with bilateral pulmonary cystic lesions. Biopsy demonstrated benign neurofibroma, and genetic testing confirmed a mutation in the NF1 gene. Unfortunately, the patient died intraoperatively during an attempted surgical resection.

## Image en médecine

La neurofibromatose de type 1 (NF1) est une maladie génétique autosomique dominante qui touche environ 1/3000 naissances. Elle se manifeste principalement par des signes cutanés (taches café au lait, neurofibromes, lentigines axillaires ou inguinales). Les atteintes pulmonaires sont rares (5 à 20%), et peuvent inclure des lésions kystiques ou infiltratives. Nous rapportons l'observation d'un garçon de 13 ans, sans antécédents particuliers, qui a consulté pour dyspnée d'effort, douleurs thoraciques, asthénie et toux sèche. L'examen clinique révélait des taches café au lait multiples (>10), des lentigines axillaires et une scoliose dorsale. La radiographie thoracique objectivait une masse médiastinale postérieure droite. La tomodensitométrie (TDM) confirmait une volumineuse masse médiastinale (13 x 15 x 12cm) associée à des lésions kystiques pulmonaires bilatérales. La biopsie mettait en évidence un neurofibrome bénin, et l'étude génétique confirmait une mutation du gène NF1. Le patient est malheureusement décédé en peropératoire lors de la tentative de résection chirurgicale.

**Figure 1:**
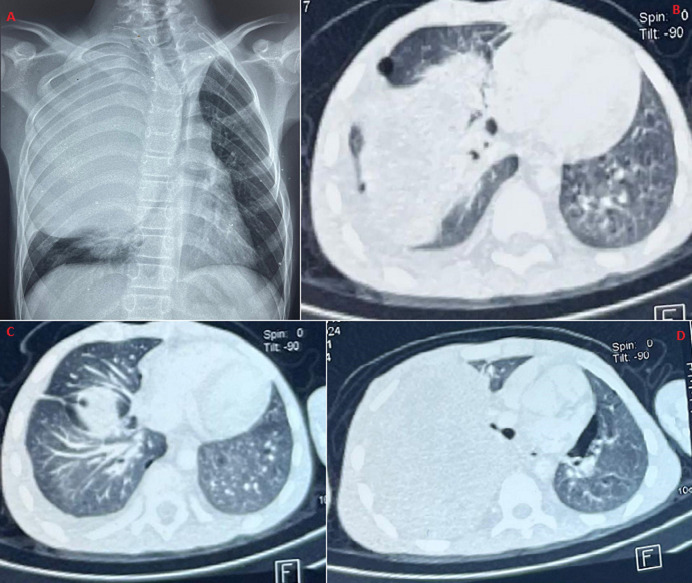
A) opacité médiastinale postérieure droite (vue de face); B) TDM thoracique en fenêtre médiastinale montrant une masse médiastinale postérieure droite, bien limitée et hétérogène, confirmant son caractère compressif; C) TDM thoracique (coupe parenchymateuse), multiples lésions kystiques pulmonaires bilatérales à paroi fine, associées à la masse médiastinale; D) TDM thoracique (coupe axiale), volumineuse de masse médiastinale droite (13 x 15 x 12cm), bien limitée, hypo dense hétérogène, infiltrant le plexus intercostal

